# Inhibitory effect of oestradiol on the cardiac K_V_7.1/KCNE1 channel is species dependent

**DOI:** 10.1113/EP092531

**Published:** 2025-05-05

**Authors:** Veronika A. Linhart, Lucas Dauga, Sara I. Liin

**Affiliations:** ^1^ Department of Biomedical and Clinical Sciences Linköping University Linköping Sweden

**Keywords:** electrophysiology, hormone, KCNQ1

## Abstract

Oestradiol (17β‐E2) is reported to prolong the cardiac action potential duration and QT interval, in part by affecting cardiac ion channels. Previous studies found inhibiting 17β‐E2 effects on the repolarizating cardiac K_V_7.1/KCNE1 channel, or its native current, in heterologous expression systems or tissue from animal species. However, there is variability in reported 17β‐E2 effects and required concentrations. In this work, we aimed to test whether a contributing factor may be different pharmacological profiles of K_V_7.1/KCNE1 channels from different species. To this end, we used the two‐electrode voltage clamp technique to characterize and quantitatively compare the effects of 17β‐E2 on K_V_7.1/KCNE1 channels from guinea pig, zebrafish, and rabbit expressed in *Xenopus* oocytes. We found that K_V_7.1/KCNE1 of all tested species is inhibited by 17β‐E2, although with species variability in the response. The guinea pig channel responded similar to previous reports for the human channel with a concentration‐dependent reduction in the overall conductance. In contrast, the rabbit channel was sensitive to lower 17β‐E2 concentrations, whereas the zebrafish channel responded with an additional inhibiting effect seen as a shifted voltage dependence of channel opening toward more positive voltages. By testing the 17β‐E2 response of K_V_7.1 alone, and by combining K_V_7.1 and KCNE1 subunits from different species, we conclude that the species variability is not simply dictated by one of the subunits but rather by the K_V_7.1/KCNE1 complex. The species variability in the 17β‐E2 response of K_V_7.1/KCNE1 could be considered when choosing appropriate animal models or interpreting findings from different experimental models.

## Abstract (highlights)

- **What is the central question of this study?**
  Does oestradiol (17β‐E2) affect the K_V_7.1/KCNE1 channel from different species used in hormone research similarly to the human K_V_7.1/KCNE1 channel?
- **What is the main finding and its importance?**
  17β‐E2 inhibited channels from all tested species, with the guinea pig channel responding similarly to that previously reported for human K_V_7.1/KCNE1, the rabbit channel being more sensitive to lower concentrations, and the zebrafish channel responding also with a shifted voltage dependence of channel opening. These findings can guide the selection of appropriate animal models and improve interpretation of studies on 17β‐E2 cardiac effects.

## 1 INTRODUCTION

Ion channels are important for the cardiac action potential. Inward flow of Na^+^ and Ca^2+^ result in depolarizing currents, while outward flow of K^+^ results in repolarization (Hedley et al., 2009; Nerbonne & Kass, 2005). The various ionic currents are required for action potential propagation and cardiomyocyte contraction, and for their synchronization. Altered activity of any of the ion channels involved may result in cardiac arrythmias or even sudden cardiac death (Hedley et al., 2009; Nerbonne & Kass, 2005; Rodriguez et al., 2001). For instance, a prolonged QT interval on the electrocardiogram, also known as long QT syndrome (LQTS), can be the result of altered activity in cardiac ion channels (Nakano & Shimizu, 2016).

LQTS may be either congenital or acquired. Over 90% of congenital LQTS cases are due to mutations in the genes encoding three different cardiac ion channels, namely, K_V_7.1/KCNE1, hERG (underlying the slow, *I*_Ks_, and rapid, *I*_Kr_, component of the delayed rectifier K^+^ current, respectively) and Na_V_1.5 (Guettler et al., 2019). Acquired LQTS can be caused by drug therapy or other endogenous and exogenous factors, modulating the activity of cardiac ion channels. Many examples of drugs which are well recognized contributors to or causes of the prolonged QT interval can be found in the literature, including, for example, numerous antibiotics, antiarrhythmic drugs and opiates (Cubeddu, 2016; Kannankeril et al., 2010). Some of these drugs, such as chromanol 293B, inhibit *I*_Ks_ and contribute to LQTS (Cubeddu, 2016). Additionally, specific steroid sex hormones are reported to affect the QT interval, where testosterone and progesterone generally induce QT‐shortening effects whereas the main oestrogen, oestradiol (17β‐E2), is considered to have QT‐prolonging effects (reviewed in Asatryan et al., 2021). The deviating QT‐shortening and QT‐prolonging effects of testosterone and 17β‐E2 in men and women, respectively, has been suggested to contribute to a longer heart rate‐corrected QT interval and increased risk of specific arrhythmias in women (Asatryan et al., 2021; Odening & Koren, 2014). For women with LQT1 (affecting *I*_Ks_), there is decreased risk of cardiac events after the onset of menopause (Buber et al., 2011).

One of the cardiac ion channels reported to be affected by 17β‐E2 is the K_V_7.1/KCNE1 channel, which is composed of four K_V_7.1 subunits and one to four KCNE1 subunits (Sanguinetti et al., 1996; Wang & Kass, 2012). 17β‐E2 inhibits the human K_V_7.1/KCNE1 channel heterologously expressed in Chinese hamster ovary (CHO) cells or *Xenopus laevis* oocytes (Moller & Netzer, 2006). In our recent study of human K_V_7.1/KCNE1 expressed in *Xenopus* oocytes, we found that 17β‐E2 reduces the overall channel conductance in a concentration‐dependent manner, without altering the voltage dependence of channel opening (Erlandsdotter et al., 2023). Moreover, 17β‐E2 has been shown to inhibit the native *I*_Ks_ in guinea pig (Tanabe et al., 1999) and rabbit (Erlandsdotter et al., 2023) cardiomyocytes, and to downregulate mRNA levels of the K_V_7.1/KCNE1 channel in rabbit cardiac tissue (Drici et al., 1996). Despite the extensive literature on pro‐arrhythmic effects of 17β‐E2, there are conflicting data on which 17β‐E2 concentrations are needed to induce effects on cardiac ion channels like K_V_7.1/KCNE1 and hERG (e.g., Kauthale et al., 2015; Kurokawa et al., 2008; Moller & Netzer, 2006), and the 17β‐E2 effects on action potential duration or QT interval vary between studies (e.g., Cheng et al., 2012; Odening et al., 2012; Prajapati et al., 2022; Tanabe et al., 1999). Theoretically, several factors may contribute to discrepancies in the literature, such as whether acute or long‐term 17β‐E2 exposure is assessed, differential roles of affected ion channel(s) in different cell types or species, and/or different pharmacological profiles of ion channels from different species (Tanner & Beeton, 2018).

In this study, we aim to provide insights into whether there are differences in the pharmacological response of the K_V_7.1/KCNE1 channel from different species to 17β‐E2. To allow for a side‐by‐side characterization of the 17β‐E2 response in a well‐controlled experimental system, we heterologously expressed K_V_7.1/KCNE1 from species commonly used to study cardiac electrophysiology in *Xenopus* oocytes, and performed similar 17β‐E2 experiments to our recent study on human K_V_7.1/KCNE1 expressed in *Xenopus* oocytes (Erlandsdotter et al., 2023). We focused on K_V_7.1/KCNE1 from guinea pig, rabbit and zebrafish, motivated by the previous use of guinea pig and rabbit in hormone studies, the overall similar role of *I*_Ks_ in ventricular repolarization in human, guinea pig and rabbit cardiomyocytes (Hoppe et al., 2001; Hornyik et al., 2020), and the emerging role of zebrafish for studies of *I*_Ks_ and cardiac electrophysiology (De la Cruz et al., 2023; Ravens, 2018; Simpson et al., 2020; Vornanen & Hassinen, 2016). The contribution of *I*_Ks_ to cardiomyocyte repolarization in these species is in contrast to mouse and rat, in which repolarization is driven by other K^+^ currents (Joukar, 2021).

We found that the guinea pig K_V_7.1/KCNE1 channel shows a similar 17β‐E2 response to that of the human K_V_7.1/KCNE1 channel, seen as a concentration‐dependent reduction in overall channel conductance that occurred at comparable 17β‐E2 concentrations as for the human channel. In contrast, the rabbit K_V_7.1/KCNE1 channel responded to notably lower 17β‐E2 concentrations, whereas the zebrafish K_V_7.1/KCNE1 channel responded to similar 17β‐E2 concentrations as the human K_V_7.1/KCNE1 channel but with an additional inhibitory response seen as a shifted voltage dependence of channel opening to more positive voltages. Altogether, this study demonstrates species‐dependent differences in K_V_7.1/KCNE1 channel responses to 17β‐E2, which may aid in the interpretation of 17β‐E2 animal studies, and could help guide the most suitable animal models for oestrogen hormone research and facilitate the translation of findings across different experimental models.

## 2 METHODS

### 2.1 Ethical approval

To isolate oocytes, female *X. laevis* frogs were used (from Nasco International, Fort Atkinson, WI, USA). The surgical procedure was approved by the regional ethics board in Linköping, Sweden (Case no. 1941 and 14515), and conforms to national and international guidelines (Directive 2010/63/EU of the European Parliament on the protection of animals used for scientific purposes). The investigators understand the ethical principles under which the journal operates and the work complies with the animal ethics checklist. Adult *X. laevis* frogs (10–12 cm) of age 24–55 months were used. The frog colony consisted of a total of 30 animals. The animals were housed in groups in water tanks with enrichment at the university animal facility. The water temperature was kept at 17–19°C, pH set to 6.4–7.8, and conductivity at 400–1000 µS. The room had a 12‐h light–dark cycle, with lights turned on at 07.00 h. The frogs were fed twice a week with Aquatic 4 (E) or Xenopro (Xenopus 1 Corp, Dexter, MI, USA) and monitored daily. Only female *X. laevis* frogs were used, as only female animals produce oocytes. Animals were anaesthetized by submersion for 15 min in a HEPES‐buffered (pH 7.4) bath of 1.4 g/L MS‐222 Sandoz (ethyl 3‐aminobenzoate methanesulfonate, Sigma‐Aldrich, Stockholm, Sweden). The depth of anaesthesia was monitored by checking for reflexes when the paws were pinched. During surgery, a small incision (1 cm) was made on the lower part of the abdomen to access oocyte lobes. Lobes were removed using surgical scissors. After the surgery, the small incision was sutured and local analgesics were administered to minimize pain (5 mg/mL bupivacaine and 2% xylocain gel, Distansapoteket, Stockholm, Sweden). Each animal underwent a maximum of six surgeries, with a minimal recovery time of 2 months in‐between surgeries (which alternated between the right and left side of the abdomen). The humane endpoints were euthanasia by decapitation under deep anaesthesia if any animals were not thriving or were sick. Additionally, animals were euthanized using the same approach in association with the sixth surgery or after being housed a maximum of 5 years at the animal facility. Note that experiments were performed only on isolated oocytes (no in vivo experiments were performed), and hence, no randomization or blinding of animals occurred.

### 2.2 Chemicals

All chemicals were from Sigma‐Aldrich (Stockholm, Sweden), unless stated otherwise. The experimental procedures and protocols used were conducted as described in Erlandsdotter et al. (2023), except for the following modification: the 17β‐E2 stock solution was prepared in 99.5% ethanol to a concentration of 25 mM for all experiments.

### 2.3 Molecular biology and oocyte preparation

Plasmids harbouring human or animal K_V_7.1/KCNE1 channels were cut with specific restriction enzymes and then transcribed into complementary RNA (cRNA) using the T7 mMessage mMachine transcription kit from Thermo Fisher Scientific (Stockholm, Sweden). To generate mutant channels, point mutations were introduced using the QuikChange II XL site‐directed mutagenesis PCR kit from Agilent Technologies (Kista, Sweden), with subsequent confirmation by sequencing at the Linköping University Core Facility for Molecular Biology. After isolation of ovarian lobes, lobes were dissected into smaller clusters of oocytes, treated with Liberase^TM^ to enzymatically remove residual connective tissue and follicular sheaths, and stored in sterile‐filtered modified Barth's solution (containing in mM: 88 NaCl, 1 KCl, 2.4 NaHCO_3_, 0.33 Ca(NO_3_)_2_, 0.41 CaCl_2_, 0.82 MgSO_4_, 15 HEPES and 2.5 pyruvate), with the pH adjusted to 7.6 using NaOH.

Oocytes were injected with 50 nL of K_V_7.1 or K_V_7.1/KCNE1 for each species as indicated in each results section (see Table 1 for accession number and Table 2 for ng used in each injection). To account for differences in codon optimization and expression efficiency across species, RNA concentrations were adjusted to achieve proper‐sized currents for experiments and currents with robust K_V_7.1/KCNE1 behaviour. We typically maintained the K_V_7.1:KCNE1 ratio used previously for the human K_V_7.1/KCNE1 channel (Erlandsdotter et al., 2023), which ensures an excess of the smaller auxiliary KCNE1 subunit promoting a saturating KCNE1 stoichiometry of assembled K_V_7.1/KCNE1 channels. However, for guinea pig K_V_7.1/KCNE1, a higher amount of KCNE1 RNA was used to ensure sufficient excess of KCNE1, as less KCNE1 generated some oocytes with currents indicating little or no KCNE1 co‐assembly. Note that later 17β‐E2 effects were determined as relative effects within each oocyte (i.e., related to each intrinsic control recording). Hence, the adjusted amounts of RNA did not contribute to species variability in the 17β‐E2 responses, but rather ensured robust K_V_7.1/KCNE1 assembly and proper‐sized currents. Injected oocytes were incubated in MBS for 2 days at 16°C, or alternatively for 1–2 days at 8°C, until electrophysiological measurements were performed.

**TABLE 1:** Summary of GenBank accession no. for the constructs used.

Species	Subunit	GenBank accession no.
Human	K_V_7.1	NM_000218
Human	KCNE1	NM_000219
Guinea pig	K_V_7.1	NM_001172821.1
Guinea pig	KCNE1	NM_001172972.1
Zebrafish	K_V_7.1	ENSDART00000083514
Zebrafish	KCNE1	XM_021479085
Rabbit	K_V_7.1	XM_008252197.2
Rabbit	KCNE1	NM_001109822

**TABLE 2:** Summary of amount of RNA used in injections.

Species	K_V_7.1 when injected alone (ng)	K_V_7.1 when co‐injected with KCNE1 (ng)	KCNE1 (ng)
Human	25	12.5	7.5
Guinea pig	2.5	1.25	1.25
Zebrafish	0.5	0.25	0.15
Rabbit	1.8	0.9	0.55

### 2.4 Two‐electrode voltage clamp experiments on *Xenopus* oocytes

Two‐electrode voltage clamp experiments were performed on a Dagan CA‐1B amplifier (Dagan, Minneapolis, MN, USA) or an AxoClamp 900A amplifier (Molecular Devices, San Jose, CA, USA). The oocyte chamber was continuously perfused at a speed of 1 mL/min using a Minipuls 3 peristaltic pump (Gilson, Middleton, WI, USA) with an extracellular recording solution containing (in mM): 88 NaCl, 1 KCl, 0.4 CaCl_2_, 0.8 MgCl_2_ and 15 HEPES, with the pH adjusted to 7.4 using NaOH. Experiments performed in this extracellular solution are referred to as ‘control’. Whenever the extracellular solution was supplemented with 17β‐E2 or vehicle (ethanol) only, this is indicated in the text and figures/tables. 17β‐E2 (or vehicle only) was diluted to indicated concentrations in the extracellular solution and applied extracellularly using the pump until a stable effect on current amplitude was observed, achieved after approximately 5 min (or applied for a minimum of 7 min if no effect was observed), monitored by running an application protocol stepping from a holding voltage of –80 mV to a test voltage of +40 mV every 10 s. 17β‐E2 concentrations were chosen to allow direct comparison with Erlandsdotter et al. (2023).

Most experiments were conducted using a standard voltage clamp protocol, with the holding voltage set to −80 mV. K_V_7.1/KCNE1 channel activation was induced by incremental depolarizing steps of 10 mV, stepping from −80 mV to +80 mV. Each test pulse was followed by a −30 mV tail pulse, which generated tail currents. The protocol used to study the zebrafish K_V_7.1/KCNE1 channel had a pre‐pulse to −120 mV and the holding voltage set to −100 mV. The activation was induced by incremental depolarizing steps of 10 mV, stepping from −100 to +90 mV. All experiments were performed at room temperature (approximately 20°C).

### 2.5 Electrophysiological analysis

To quantify the voltage dependence of channel opening, tail currents were measured shortly after stepping to the tail voltage and plotted against the preceding activation voltage. A Boltzmann function was fitted to the data to generate the conductance versus voltage (*G*(*V*)) curve:

$$\;G\left(V\right)=G_{\min}+\frac{\left(G_{\max}-G_{\min}\right)}{\left[1+\exp\left(\frac{V_{50-x}}{s}\right)\right]}$$

where *G*_min_ and *G*_max_ refer to the minimum and maximum conductance, respectively, *V*_50_ refers to the midpoint of the curve, and *s* refers to the slope of the curve. Table 3 summarizes the biophysical properties of the constructs used. Current amplitude at +40 mV (*I*_40mV_) was quantified at the end of the activation pulse to +40 mV. The change in *G*_max_, *V*_50_ or *I*_40mV_ in each oocyte was used to quantify the compound effect on each parameter. For experiments where currents or conductance did not clearly show signs of saturation, the fits should be considered as an approximation.

**TABLE 3:** Intrinsic properties of studied channels.

Construct	*V*_50_ Mean ± SEM [± SD] (mV)	*s* Mean ± SEM [± SD] (mV)	*n* [*N*]
Human K_V_7.1 WT	−25.0 ± 1.3 [± 4.7]	9.2 ± 1.1 [± 4.0]	13 [>5*]
Human K_V_7.1/KCNE1 WT	+30.1 ± 1.0 [± 12.2]	13.7 ± 0.3 [± 1.6]	30–152 [>5*]
Guinea pig K_V_7.1/KCNE1 WT	+38.0 ± 1.6 [± 5.1]	16.1 ± 0.3 [± 1.9]	41 [>5*]
Zebrafish K_V_7.1 WT	−48.3 ± 3.3 [± 9.9]	17.1 ± 1.7 [± 5.1]	9 [3]
Zebrafish K_V_7.1/KCNE1 WT	+39.7 ± 3.5 [± 17.6]	21.2 ± 1.0 [± 4.9]	26 [8]
Rabbit K_V_7.1 WT	−25.9 ± 5.3 [± 12.9]	13.2 ± 0.3 [± 0.8]	6 [1]
Rabbit K_V_7.1/KCNE1 WT	+23.3 ± 2.0 [± 13.1]	17.8 ± 0.4 [± 2.5]	43 [> 5*]
			
Human K_V_7.1/KCNE1_K69Q	+33.4 ± 3.1 [± 11.3]	12.5 ± 0.6 [± 2.1]	13 [3]
Human K_V_7.1 + Rabbit KCNE1	+27.4 ± 2.3 [± 11.1]	16.0 ± 0.5 [± 2.6]	24 [4]
Rabbit K_V_7.1 + Human KCNE1	+20.3 ± 1.2 [± 5.9]	14.1 ± 0.4 [± 2.0]	24 [8]

*Note*: *V*_50_ and *s* were determined from Boltzmann fits, as described in the methods section. *n* denotes the number of oocytes, *N* denotes the number of frogs used. >5* indicates that at least five frogs were used for pooled or previously published data. Data for human K_V_7.1 and K_V_7.1/KCNE1 are included for comparison and duplicated from Erlandsdotter et al. (2023) and Hiniesto‐Inigo et al. (2023, 2024). Some of the observations for guinea pig and rabbit K_V_7.1/KCNE1 were also included in descriptive data in Hiniesto‐Inigo et al. (2024).

To plot the concentration dependence of the compound‐induced effect as a function of the compound concentration, the following concentration–response curve was fitted to the data:

$$R=\frac{R_{\mathrm{MAX}}}{\left[1+{\left(\frac{EC_{50}}{C}\right)}^{N}\right]}$$

where *R* is the compound response (either Δ*G*_MAX_ or Δ*I*_40mV_), *R*_MAX_ is the maximum response, EC_50_ refers to the concentration required to reach 50% of maximum response, *C* is the concentration of the test compound and *N* is the Hill coefficient. It should be noted that not all curves could be fitted with high confidence. In such cases, no fit was performed, as indicated by the term ‘ambiguous’ in the figure legend.

### 2.6 Statistical analysis

Average values are shown as means ± SD. SD is used in figures while SEM is included in the related Tables to allow for comparison with other studies in the field using similar experimental approaches, for which data commonly are shown as means ± SEM. No blinding was applied as only one treatment was assessed. When comparing two groups, one‐way ANOVA followed by Šidák’s multiple comparisons test was used. A one‐sample Student's *t*‐test was used when comparing the observed effects to a hypothetical effect of 0 (0 mV for Δ*V*_50_, 0% for Δ*G*_max_). Statistical analysis of data was performed using GraphPad Prism 9 (GraphPad Software, Boston, MA, USA). *n* represents the number of oocytes, and *N* denotes the number of frogs used. These values are indicated in the figure legends and tables throughout the paper. If *n* < 30, all data points are plotted in the figures.

## 3 RESULTS

### 3.1 17β‐E2 inhibits K_V_7.1/KCNE1 channels of different species

**FIGURE 1:**
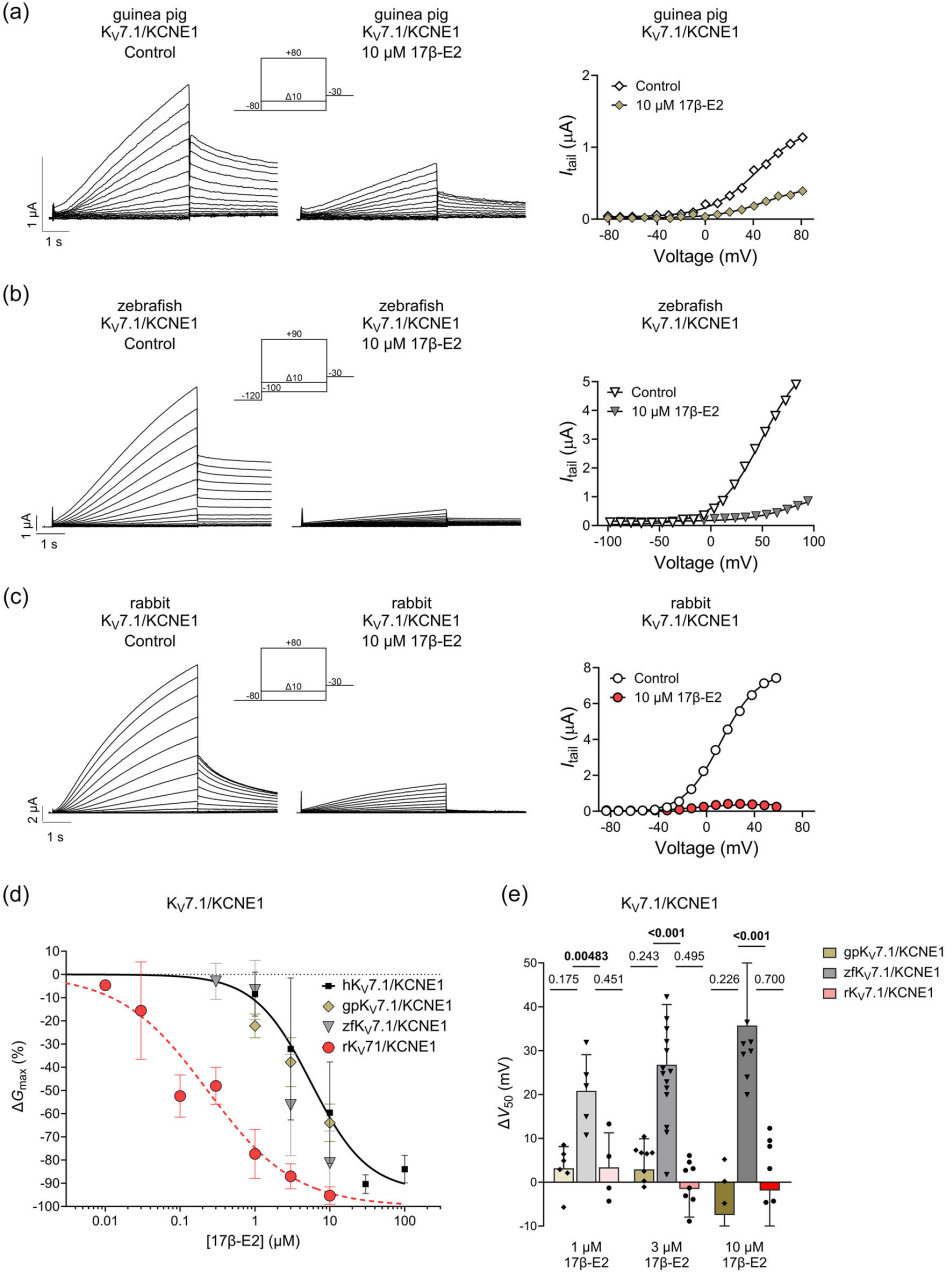
17β‐E2 inhibits the K_V_7.1/KCNE1 channel of different species. (a) Representative current family with corresponding *G*(*V*) curve of guinea pig K_V_7.1/KCNE1 in the absence (left) and presence (middle) of 10 µM 17β‐E2. The voltage protocol used is shown as inset. Curves in the *G*(*V*) plot (right) represent Boltzmann fits (see Methods for details). For this representative cell, *V*_50,Ctrl_ = +43.1 mV, *I*_tailmax,Ctrl _= 1.3 µA, *V*_50,17β‐E2_ = +50.1 mV and *I*_tailmax,17β‐E2_ = 0.5 µA. (b) Same as (a) but for the zebrafish K_V_7.1/KCNE1 channel. For this representative cell, *V*_50,Ctrl_ = +49.7 mV, *I*_tailmax,Ctrl _= 6.0 µA, *V*_50,17β‐E2_ = +81.7 mV and *I*_tailmax,17β‐E2_ = 1.2 µA. (c) Same as (a) but for the rabbit K_V_7.1/KCNE1 channel. For this representative cell, *V*_50,Ctrl_ = +11.9 mV, *I*_tailmax,Ctrl _= 7.8 µA, *V*_50,17β‐E2_ = −11.1 mV and *I*_tailmax,17β‐E2_ = 0.4 µA. (d) Concentration dependence of the 17β‐E2 effect on *G*_max_ of K_V_7.1/KCNE1 of indicated species. See Methods for details of the concentration‐response fit. Best fit for rabbit: maximum ∆*G*_max_ = −100% and EC_50_ = 0.23 µM. Guinea pig and zebrafish were ambiguous. Data are shown as means ± SD (with SEM shown in Table 6). *n* = 4–19, *N* = 1–6. Small error bars are covered by symbols. Human data are included for comparison and duplicated from Erlandsdotter et al. (2023) (best fit for human: maximum ∆*G*_max_ = −94% and EC_50_ = 5.6 µM). (e) Summary of the effect of 1, 3 and 10 µM 17β‐E2 on K_V_7.1/KCNE1 from guinea pig, zebrafish and rabbit on the voltage dependence of channel activation. Data are shown as means ± SD (with SEM shown in Table 6). *n* = 4–15, *N* = 1–6. Statistics denote one‐sample *t*‐test comparing to a hypothetical value of 0.

To allow for a comparison of the 17β‐E2 response of K_V_7.1/KCNE1 from different species with the response previously observed in the human K_V_7.1/KCNE1 channel (Erlandsdotter et al., 2023), we expressed the K_V_7.1/KCNE1 channel from guinea pig, zebrafish or rabbit in *X. laevis* oocytes and tested the effect of different concentrations of 17β‐E2. Table 3 summarizes the intrinsic properties of the assessed channels, with representative examples under control conditions shown in Figure 1. As has been previously described (Hiniesto‐Inigo et al., 2024), the voltage dependence of channel opening of guinea pig and rabbit K_V_7.1/KCNE1 is shifted about 7–8 mV to more positive or negative voltages, respectively, compared to human K_V_7.1/KCNE1 (Table 3). The zebrafish K_V_7.1/KCNE1 displayed a shifted voltage dependence of channel opening of about 9 mV towards more positive voltages, compared to human K_V_7.1/KCNE1 (Table 3). Additionally, the zebrafish channel closed slowly (Figure 1b). To facilitate channel closure in‐between each current sweep of the zebrafish channel, we included a pre‐pulse to –120 mV (see Methods). To assess the 17β‐E2‐mediated response, we quantified 17β‐E2 induced alterations in the maximum conductance (*G*_max_) and the voltage required for achieving half‐maximal conductance (*V*_50_). These measurements provided insights into the modulatory influence of 17β‐E2 on the overall current amplitude/conductance and the voltage dependence of channel activation (see Methods for details). Under these experimental conditions, there were no time‐dependent effects in the absence of 17β‐E2 (Table 4). Moreover, there were no, or only minor, effects of the highest vehicle (ethanol) concentration used in subsequent experiments (Table 5).

**TABLE 4:** Time matched controls for WT K_V_7.1/KCNE1 constructs.

	Δ*V*_50_		Δ*G*_max_		
Construct	Mean ± SEM [± SD] (mV)	*P*	Mean ± SEM [± SD] (%)	*P*	*n* [*N*]
Guinea pig K_V_7.1/KCNE1	−3.4 ± 2.0 [± 5.6]	> 0.05	+19.7 ± 6.9 [± 19.7]	> 0.05	8 [3]
Zebrafish K_V_7.1/KCNE1	+0.0 ± 1.4 [± 2.7]	> 0.05	+2.7 ± 3.9 [± 7.8]	> 0.05	4 [1]
Rabbit K_V_7.1/KCNE1	+3.6 ± 2.7 [± 4.6]	> 0.05	+12.1 ± 8.7 [± 15.2]	> 0.05	3 [2]

*Note*: *V*_50_ and *G*_max_ were determined from Boltzmann fits, as described in Methods. *n* denotes the number of oocytes, *N* denotes the number of frogs used. *P*‐values represent one‐sample *t*‐test compared to a hypothetical value of 0.

**TABLE 5:** Vehicle control experiments for WT Kv7.1/KCNE1 constructs for the vehicle ethanol equivalent to 10 µM 17β‐E2 used in experiments.

	Δ*V*_50_		Δ*G*_max_		
Construct	Mean ± SEM [± SD] (mV)	*P*	Mean ± SEM [± SD] (%)	*P*	*n* [*N*]
Guinea pig K_V_7.1/KCNE1	−3.1 ± 1.6 [± 3.9]	> 0.05	+25.4 ± 10.3 [± 25.2]	> 0.05	6 [2]
Zebrafish K_V_7.1/KCNE1	+2.9 ± 2.9 [± 5.0]	> 0.05	−7.5 ± 1.3 [± 2.3]	**< 0.05**	3 [2]
Rabbit K_V_7.1/KCNE1	−3.7 ± 1.4 [± 3.4]	**< 0.05**	+27.8 ± 1.7 [± 3.8]	**< 0.0001**	5 [2]

*Note*: *V*_50_ and *G*_max_ were determined from Boltzmann fits, as described in Methods. *n* denotes the number of oocytes, *N* denotes the number of frogs used. *P*‐values represent one‐sample *t*‐test compared to a hypothetical value of 0. *P*‐values in bold indicate statistical significance.

We found that the inhibitory effect of 17β‐E2 on the K_V_7.1/KCNE1 channel exhibited species variation (see Figure 1a–c for representative examples). For the guinea pig K_V_7.1/KCNE1 channel, we observed an inhibitory 17β‐E2 response closely resembling the response previously observed for the human K_V_7.1/KCNE1 channel. At concentrations of 1, 3 and 10 µM, 17β‐E2 reduced *G*_max_ of the guinea pig K_V_7.1/KCNE1 channel by −22.2 ± 5.1% (*P* = 0.0006), −37.8 ± 10.6% (*P* < 0.0001) and −60.0 ± 8.1% (*P* < 0.0001), respectively (Figure 1d, Table 6). Similar to the effect on the human K_V_7.1/KCNE1 channel, there was no significant shift in *V*_50_ (Figure 1e, Table 6). Therefore, the observed inhibitory effect of 17β‐E2 on the guinea pig K_V_7.1/KCNE1 closely parallels that of the human K_V_7.1/KCNE1 channel.

**TABLE 6:** Summary of the effect of indicated 17β‐E2 concentrations on listed constructs and parameters.

Construct		Δ*V*_50_		Δ*G*_max_		*n* [*N*]
	**[17β‐E2] (µM)**	**Mean** ± **SEM** **[± SD] (mV)**	***P***	**Mean** ± **SEM** **[± SD] (%)**	***P***	
Guinea pig K_V_7.1/KCNE1 WT	1	+3.2 ± 2.0 [± 4.9]	> 0.05	−22.2 ± 2.3 [± 5.1]	**< 0.001**	5–6 [1]
	3	+3.0 ± 2.3 [± 7.0]	> 0.05	−37.8 ± 3.7 [± 10.6]	**< 0.0001**	8–9 [4]
	10	−7.5 ± 5.2 [± 11.7]	> 0.05	−63.9 ± 3.6 [± 8.1]	**< 0.0001**	5 [2]
Zebrafish K_V_7.1 WT	3	+3.7 ± 1.4 [± 4.1]	**< 0.05**	+9.9 ± 1.9 [± 5.8]	**< 0.001**	9 [3]
	10	+3.1 ± 1.7 [± 5.2]	> 0.05	+15.1 ± 3.2 [± 9.7]	**< 0.01**	9 [3]
Zebrafish K_V_7.1/KCNE1 WT	0.3	+13.0 ± 2.6 [± 6.3]	**< 0.01**	−3.0 ± 3.5 [± 7.8]	> 0.05	5‐6 [2]
	1	+20.8 ± 3.7 [± 8.3]	**< 0.01**	−6.7 ± 5.7 [± 12.8]	> 0.05	5 [2]
	3	+26.8 ± 3.5 [± 13.7]	**<0.001**	−56.3 ± 5.0 [± 21.8]	**< 0.0001**	15–19 [6]
	10	+35.7 ± 4.6 [± 14.3]	**<0.001**	−81.3 ± 3.4 [± 13.9]	**< 0.0001**	9–17 [6]
Rabbit K_V_7.1 WT	3	+2.2 ± 1.5 [± 3.6]	> 0.05	−1.2 ± 2.8 [± 6.9]	> 0.05	6 [1]
	10	+2.1 ± 1.3 [± 3.3]	> 0.05	+4.6 ± 4.4 [± 10.9]	> 0.05	6 [1]
Rabbit K_V_7.1/KCNE1 WT	0.01	+8.4 ± 2.4 [± 4.9]	**< 0.05**	−4.6 ± 1.1 [± 2.2]	**< 0.05**	4 [1]
	0.03	+7.6 ± 2.2 [± 6.5]	**< 0.01**	−15.4 ± 7.9 [± 21.0]	> 0.05	7–9 [3]
	0.1	+12.0 ± 4.0 [± 8.9]	**< 0.05**	−52.4 ± 5.3 [± 9.1]	**< 0.01**	3–5 [2]
	0.3	+2.9 ± 4.7 [± 12.5]	> 0.05	−48.0 ± 3.3 [± 8.0]	**< 0.0001**	6–7 [2]
	1	+3.4 ± 3.9 [± 7.9]	> 0.05	−77.3 ± ‐5.3 [± 10.5]	**< 0.001**	4 [1]
	3	−1.5 ± 2.1 [± 6.4]	> 0.05	−87.0 ± 1.8 [± 5.4]	**< 0.0001**	9 [3]
	10	−1.9 ± 4.7 [± 13.4]	> 0.05	−95.4 ± 1.3 [± 3.8]	**< 0.0001**	8 [3]
						
Human K_V_7.1/KCNE1_K69Q	0.3	−1.1 ± 1.6 [± 4.3]	> 0.05	−12.5 ± 5.3 [± 9.1]	> 0.05	3–7 [2]
	1	−4.0 ± 3.5 [± 9.3]	> 0.05	−27.1 ± 8.6 [± 14.9]	> 0.05	3–7 [2]
	3	+4.8 ± 2.9 [± 7.1]	> 0.05	−50.1 ± 2.9 [± 7.2]	**< 0.0001**	6 [1]
	10	+5.9 ± 4.3 [± 9.7]	> 0.05	−75.4 ± 2.2 [± 4.8]	**< 0.0001**	5 [1]
Human K_V_7.1 + Rabbit KCNE1	1	+6.0 ± 2.1 [± 5.2]	**< 0.05**	−13.6 ± 2.9 [± 6.4]	**< 0.01**	5–6 [2]
	3	+7.9 ± 1.0 [± 3.4]	**<0.0001**	−39.2 ± 6.0 [± 19.0]	**< 0.001**	10–11 [4]
	10	+7.8 ± 2.0 [± 4.9]	**< 0.05**	−77.8 ± 6.2 [± 15.3]	**< 0.0001**	6 [2]
Rabbit K_V_7.1 + human KCNE1	1	+3.2 ± 1.0 [± 3.8]	**< 0.01**	−10.9 ± 2.6 [± 8.8]	**< 0.01**	12–14 [6]
	3	+4.8 ± 1.9 [± 5.7]	**< 0.05**	−67.1 ± 4.9 [± 12.9]	**< 0.0001**	7–9 [5]
	10	−6.1 ± 2.3 [± 4.7]	> 0.05	−91.9 ± 3.0 [± 6.7]	**< 0.0001**	4–5 [3]

*Note*: *V*_50_ and *G*_max_ were determined from Boltzmann fits, as described in Methods. *n* denotes the number of oocytes, *N* denotes the number of frogs used. *P*‐values denote one sample *t‐*test compared to a hypothetical value of 0. *P*‐values in bold indicate statistical significance.

17β‐E2 also induced an inhibitory effect on the zebrafish K_V_7.1/KCNE1. At concentrations of 1, 3 and 10 µM, 17β‐E2 reduced *G*_max_ of the zebrafish K_V_7.1/KCNE1 channel by −6.7 ± 12.8% (*P* = 0.3054), −56.3 ± 21.8% (*P* < 0.0001) and −81.3 ± 13.9% (*P* < 0.0001), respectively (Figure 1d, Table 6). Hence, the reduction in *G*_max_ at the highest 17β‐E2 concentrations appeared slightly more pronounced for zebrafish K_V_7.1/KCNE1 compared to the guinea pig and human channels. Because of uncertainties in determining *G*_max_ for the right‐shifted zebrafish K_V_7.1/KCNE1 channel, we also quantified the inhibitory effect of 17β‐E2 at +90 mV, which was the most positive voltage tested. This quantification gave a similar outcome as the *G*_max_ analysis at the higher concentrations, and a more pronounced reduction at 1 µM, with 1, 3 and 10 µM 17β‐E2 reducing conductance by −21.1 ± 6.8% (*P* = 0.0022), −61.8 ± 22.0% (*P* < 0.0001) and −84.1 ± 15.4% (*P* < 0.0001), respectively. Additionally, 17β‐E2 induced a rightward shift of *V*_50_ by +20.8 ± 3.7 mV (*P* = 0.0048), +26.8 ± 13.7 mV (*P* < 0.0001) and +35.7 ± 14.3 mV (*P* < 0.0001) for 1, 3 and 10 µM, respectively (Figure 1e, Table 6). Therefore, the observed inhibitory effect of 17β‐E2 on the zebrafish K_V_7.1/KCNE1 largely parallels that of the human K_V_7.1/KCNE1 channel for *G*_max_, with the addition of an inhibitory effect on *V*_50_.

For the rabbit K_V_7.1/KCNE1 channel, we observed greater sensitivity to 17β‐E2 at lower concentrations, compared to the human, guinea pig and zebrafish channels. Concentrations of 1, 3 and 10 µM led to a reduction of *G*_max_ of the rabbit K_V_7.1/KCNE1 channel by −77.4 ± 10.5% (*P* = 0.0007), −87.0 ± 5.4% (*P* < 0.0001) and −95.4 ± 3.8% (*P* < 0.0001), respectively, with pronounced *G*_max_ reduction observed also at 17β‐E2 concentrations lower than 1 µM (Figure 1d, Table 6). A modest significant rightward *V*_50_ shift was noticed for concentrations of 0.1 µM and lower (< +15 mV). However, the was no significant *V*_50_ effect at higher concentrations (Figure 1e, Table 6). Therefore, the observed inhibitory effect of 17β‐E2 on the rabbit K_V_7.1/KCNE1 occurs at lower concentrations than that of the human K_V_7.1/KCNE1 channel for *G*_max_, with the addition of an inhibitory effect on *V*_50_ at lower 17β‐E2 concentrations.

In summary, our data show that 17β‐E2 induces inhibitory effects on K_V_7.1/KCNE1 channels from different species, albeit with variations in the concentration dependence of the 17β‐E2 effect. Moreover, 17β‐E2 shifted *V*_50_ of the zebrafish K_V_7.1/KCNE1 channel to more positive voltages in a concentration‐dependent manner, an inhibiting effect not observed for human and guinea pig K_V_7.1/KCNE1, and observed only at low 17β‐E2 concentrations for rabbit K_V_7.1/KCNE1.

### 3.2 The KCNE1 subunit is of importance for the enhanced inhibitory effects of 17β‐E2 in the zebrafish and rabbit channels

**FIGURE 2:**
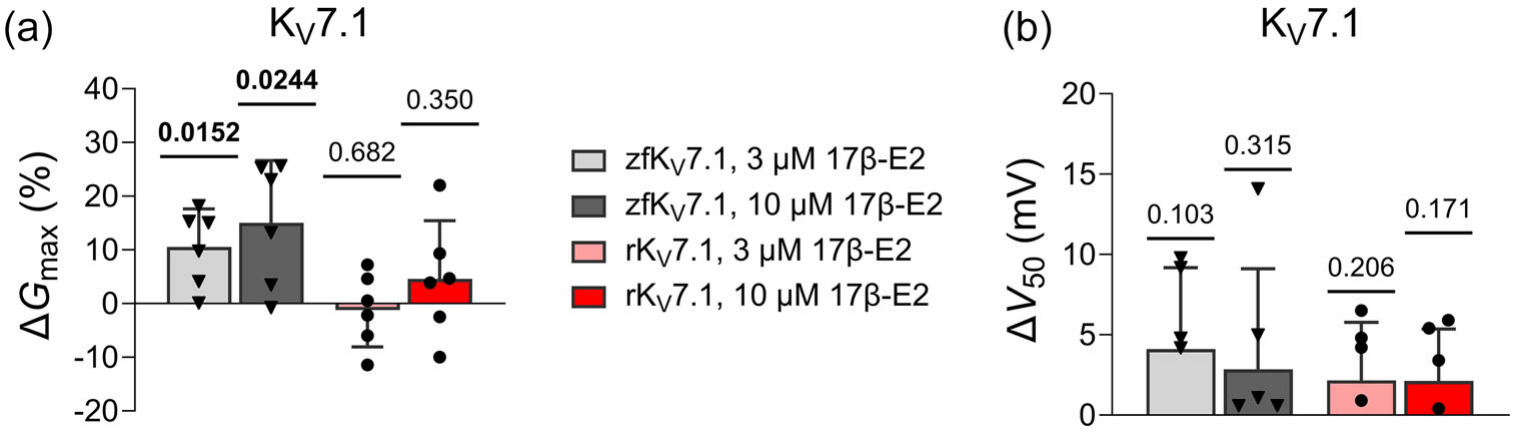
17β‐E2 does not inhibit the K_V_7.1 channel alone (no KCNE1 co‐expression) of different species. (a) Summary of the effect of 3 and 10 µM of 17β‐E2 on K_V_7.1 from zebrafish and rabbit on *G*_max_. Data shown as means ± SD (with SEM shown in Table 6). *n* = 6, *N* = 1–3. (b) Same as in (a) but for *V*_50_. Statistics denote one‐sample *t*‐test to compare to a hypothetical value of 0.

Our previous study (Erlandsdotter et al., 2023) showed that the inhibitory effect of 17β‐E2 seen on the human K_V_7.1/KCNE1 channel requires the KCNE1 subunit, and that the extent of the inhibitory effect is determined mainly by residues on the intracellular region of the KCNE1 subunit. Given the interspecies variation in the 17β‐E2 effect on zebrafish and rabbit K_V_7.1/KCNE1 compared to human K_V_7.1/KCNE1, we tested if the 17β‐E2 effect on zebrafish and rabbit K_V_7.1/KCNE1 is also dependent on the KCNE1 subunit. Similar to the human K_V_7.1 channel, 17β‐E2 concentrations up to 10 µM did not affect the *G*_max_ or *V*_50_ of rabbit Kv7.1 alone (Figure 2, Table 6; ∆*G*_max_ and ∆*V*_50_ was within ±5% and 5 mV, respectively). For zebrafish K_V_7.1 alone, 17β‐E2 did not affect *V*_50_ (∆*V*_50_ was within 4 mV) and induced only a minor increase in *G*_max_ (+15.1 ± 9.7%, *P* = 0.0016 at 10 µM of 17β‐E2) (Figure 2, Table 6). Altogether, these data show that the KCNE1 subunit is necessary for the 17β‐E2‐induced inhibition of the K_V_7.1/KCNE1 channels from zebrafish and rabbit, and that the enhanced inhibitory effects require co‐expression with the KCNE1 subunit.

### 3.3 A non‐conserved residue at position 69 in the human KCNE1 subunit is not responsible for the increased potency of 17β‐E2 seen in the rabbit K_V_7.1/KCNE1 channel

**FIGURE 3:**
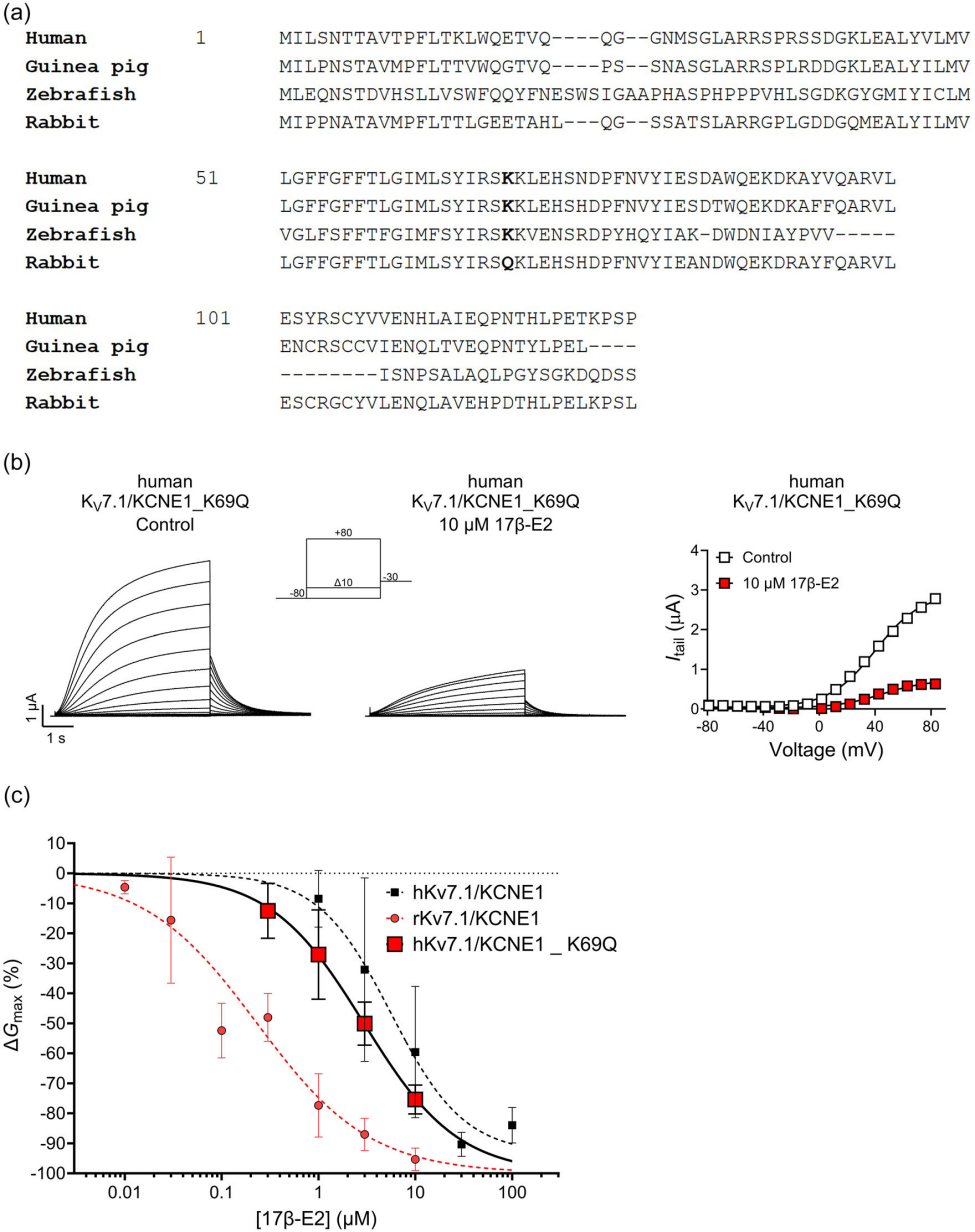
Non‐conserved amino acid at position 69 in human KCNE1 does not account for the deviating 17β‐E2 response of the rabbit K_V_7.1/KCNE1 channel. (a) Sequence alignment of KCNE1 from indicated species, performed using UniProt Align. A non‐conserved residue in rabbit KCNE1 is indicated in bold. Note that the alignment of the zebrafish construct was challenging due to non‐conserved regions. (b) Representative current family with corresponding *G*(*V*) curve of the human K_V_7.1/KCNE1_K69Q mutant channel in the absence (left) and presence (middle) of 10 µM 17β‐E2. The voltage protocol used is shown as inset. Curves in the *G*(*V*) plot (right) represent Boltzmann fits (see Methods for details). For this representative cell, *V*_50,Ctrl_ = +40.1 mV, *I*_tailmax,Ctrl _= 2.9 µA, *V*_50,17β‐E2_ = +45.2 mV and *I*_tailmax,17β‐E2_ = 0.7 µA. (c) Concentration dependence of the 17β‐E2 effect on *G*_max_ of human K_V_7.1/KCNE1_K69Q. See Methods for details of the concentration–response fit. Best fit for K69Q: maximum ∆*G*_max_ = −100% and EC_50_ = 2.9 µM. Data shown as means ± SD (with SEM shown in Table 6). *n* = 3–6, *N* = 1–2. Small error bars are covered by symbols. Data for human and rabbit WT K_V_7.1/KCNE1 from Figure 1d are included for comparison.

Our previous study (Erlandsdotter et al., 2023) suggested that a specific region of the KCNE1 C‐terminus is required for the 17β‐E2‐mediated inhibition of human K_V_7.1/KCNE1, namely, between residues 67 and 77. In this region, there is one notable difference in the KCNE1 subunit when comparing rabbit with human, guinea pig and zebrafish KCNE1. At the residue corresponding to position 69 in human KCNE1, human, guinea pig and zebrafish have a lysine, whereas the rabbit KCNE1 has a glutamine (Figure 3a). To test whether this unique glutamine in the rabbit KCNE1 subunit contributes to the ability of the rabbit K_V_7.1/KCNE1 channel to respond to 17β‐E2 at lower concentrations, we generated the human K_V_7.1/KCNE1_K69Q mutant channel to mimic the rabbit KCNE1 subunit at this specific residue. The observed reduction in *G*_max_ was −27.1 ± 14.9% (*P* = 0.0875), −50.1 ± 7.2% (*P* < 0.0001) and −75.4 ± 4.8% (*P* < 0.0001) for 1, 3 and 10 µM 17β‐E2, respectively (Figure 3b,c, Table 6). Hence, the K_V_7.1/KCNE1_K69Q *G*_max_ response largely followed the human K_V_7.1/KCNE1 wild‐type (WT) response, and not the rabbit K_V_7.1/KCNE1 WT response. This indicates that the lysine to glutamine substitution at position 69 in the KCNE1 subunit is not responsible for the enhanced 17β‐E2‐mediated inhibitory effect in the rabbit channel.

### 3.4 The combined rabbit K_V_7.1/KCNE1 channel complex is responsible for the increased potency of 17β‐E2

As neither the K_V_7.1 subunit alone nor the singular residue at position 69 in the KCNE1 subunit was responsible for the enhanced *G*_max_ response of the rabbit K_V_7.1/KCNE1 channel to low concentrations of 17β‐E2, it raised the question of whether other regions within the KCNE1 subunit were. To explore this, we co‐injected human K_V_7.1 with the rabbit KCNE1 subunit, and vice versa.

**FIGURE 4:**
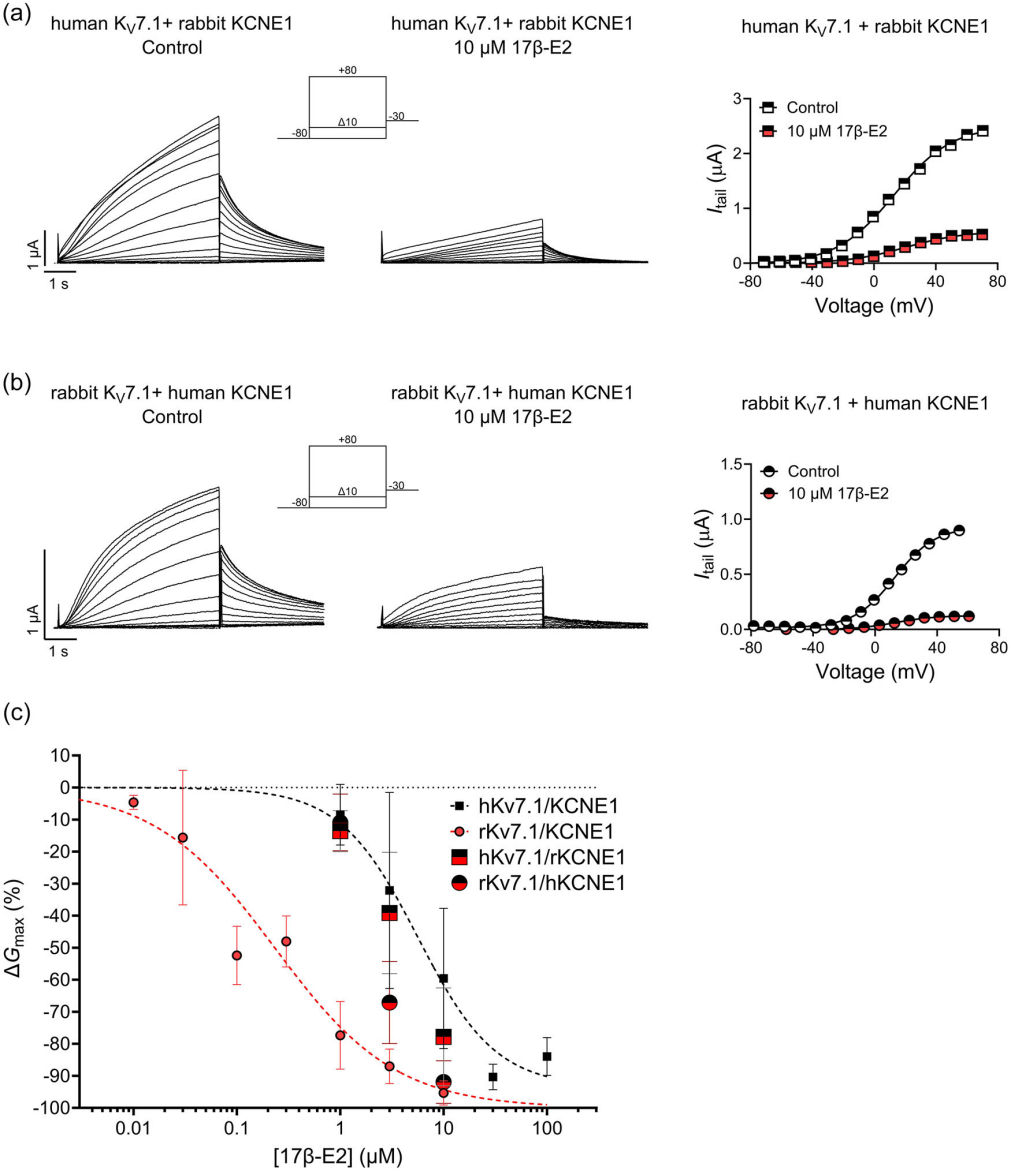
The combined K_V_7.1/KCNE1 channel complex determines the magnitude of the 17β‐E2 response of the rabbit channel. (a) Representative current family with corresponding *G*(*V*) curve of the co‐injection of human K_V_7.1 and rabbit KCNE1 in the absence (left) and presence (middle) of 10 µM 17β‐E2. The voltage protocol used is shown as inset. Curves in the *G*(*V*) plot (right) represent Boltzmann fits (see Methods for details). For this representative cell, *V*_50,Ctrl_ = +13.1 mV, *I*_tailmax,Ctrl _= 2.5 µA, *V*_50,17β‐E2_ = +20.7 mV and *I*_tailmax,17β‐E2_ = 0.6 µA. (b) Same as (a) but for rabbit K_V_7.1 and human KCNE1 co‐injection. For this representative cell, *V*_50,Ctrl_ = +12.8 mV, *I*_tailmax,Ctrl _= 0.9 µA, *V*_50,17β‐E2_ = +13.0 mV and *I*_tailmax,17β‐E2_ = 0.1 µA. (c) Concentration dependence of the 17β‐E2 effect on *G*_max_ of K_V_7.1 and KCNE1 co‐injections compared to indicated K_V_7.1/KCNE1 WT channels from Figure 1d. Data shown as means ± SD (with SEM shown in Table 6). *n* = 5–12, *N* = 1–6. Small error bars are covered by symbols. Fits for co‐injections were ambiguous.

For the human K_V_7.1/rabbit KCNE1 channel, we observed a *G*_max_ response that largely followed the human K_V_7.1/KCNE1 WT response (Figure 4a,c); 1, 3 and 10 µM 17β‐E2 reduced *G*_max_ by −13.6 ± 6.4% (*P* = 0.0089), −39.2 ± 19.0% (*P* = 0.0001) and −77.8 ± 15.3% (*P* < 0.0001), respectively, in the human K_V_7.1/rabbit KCNE1 channel (Figure 4c, Table 6). For the rabbit K_V_7.1/human KCNE1 channel, we observed a *G*_max_ response intermediate to that of the human and rabbit K_V_7.1/KCNE1 WT responses (Figure 4b,c). At 1 µM of 17β‐E2, the rabbit K_V_7.1/human KCNE1 channel demonstrated a *G*_max_ response similar to the human K_V_7.1/KCNE1 channel (Δ*G*_max_ = −10.9 ± 8.8%, *P* = 0.01; Figure 4c, Table 6), while 17β‐E2 concentrations of 3 and 10 µM led to a more prominent reduction in *G*_max_ (by −67.1 ± 12.9% and −91.9 ± 6.7%, *P* < 0.0001, respectively), approaching the response in the rabbit K_V_7.1/KCNE1 channel (Figure 4c, Table 6). In the same way, we tried to co‐express human K_V_7.1 with the zebrafish KCNE1 subunit, and vice versa, to study the origin of the *V*_50_ effect in the zebrafish channel. However, the human and zebrafish co‐expression attempts failed in generating K_V_7.1/KCNE1‐like currents, possibly indicating that the human and zebrafish subunits are unwilling to co‐assemble into functional channels under our experimental conditions.

Altogether, the findings from co‐injected human and rabbit subunits show that the magnitude of the 17β‐E2 response of K_V_7.1/KCNE1 channels from human and rabbit is not simply determined by either the K_V_7.1 subunit or the KCNE1 subunit. Rather, it appears that the K_V_7.1/KCNE1 complex is what determines the magnitude of the response.

### 3.5 Species‐dependent variability in K_V_7.1/KCNE1 channel 17β‐E2 responses translate to varying impact on current amplitude at a physiologically relevant voltage

**FIGURE 5:**
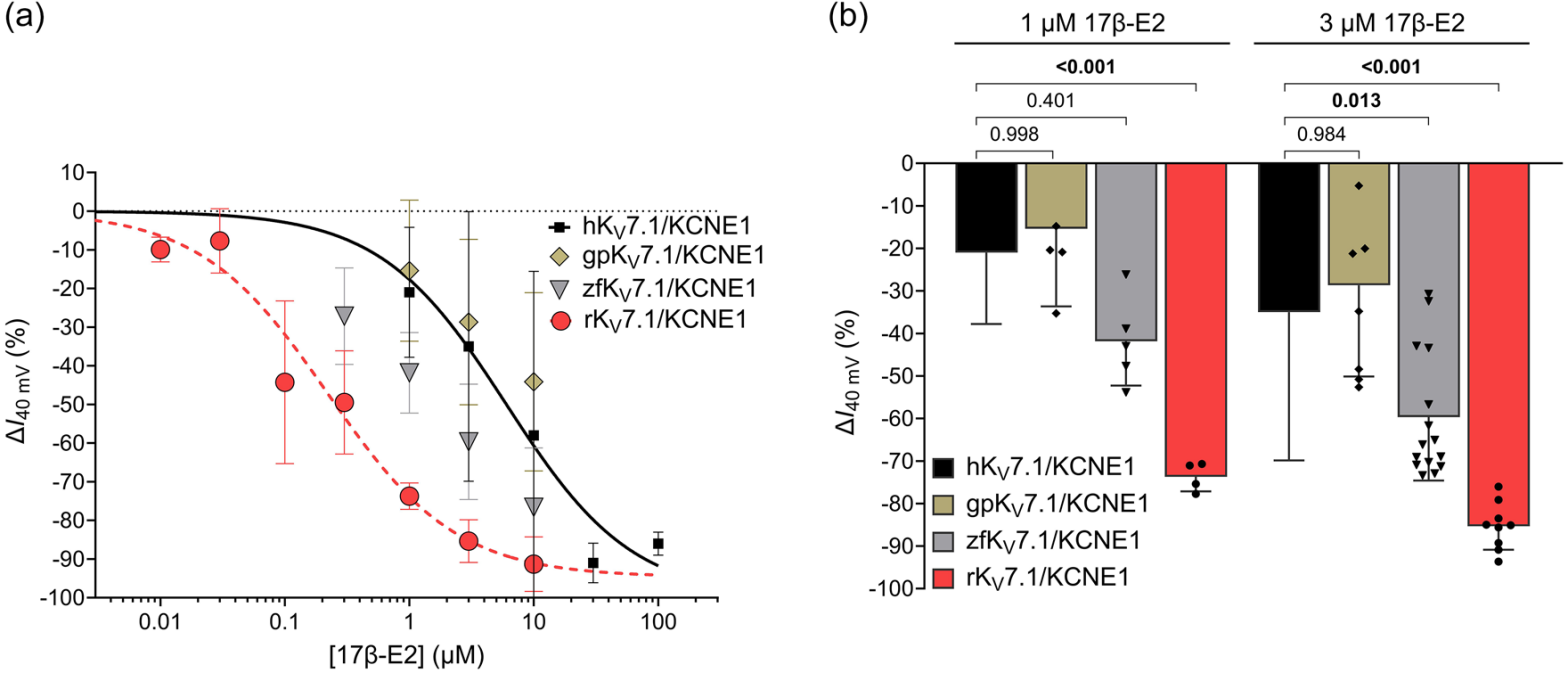
The varying 17β‐E2 responses of K_V_7.1/KCNE1 from different species translate to differing current reduction at a physiologically relevant voltage. (a) Concentration dependence of the 17β‐E2 effect on current amplitude at +40 mV of K_V_7.1/KCNE1 of indicated species. See Methods for details of the concentration–response fit. Best fit for rabbit: maximum ∆*I*_40mV_ = −94.7% and EC_50_ = 0.22 µM. Guinea pig and zebrafish were ambiguous. Data shown as means ± SD. *n* = 4−15, *N* = 4–8. Small error bars are covered by symbols. Human data are included for comparison and duplicated from Erlandsdotter et al. (2023) (Best fit for human: maximum ∆*I*_40mV_ = −100% and EC_50_ = 6.0 µM.) (b) Comparison of the ability of 1 and 3 µM of 17β‐E2 to reduce current amplitude at +40 mV of K_V_7.1/KCNE1 from indicated species. Data shown as means ± SD. *n* as in (a). Statistics denote one‐way ANOVA followed by Šidák's multiple comparisons test comparing to the effect on the human channel.

To estimate how the varying 17β‐E2 responses in K_V_7.1/KCNE1 channels from different species affect current amplitude, we quantified the 17β‐E2 effect at +40 mV (*I*_40mV_). Although this voltage is more depolarized than the typical plateau phase of the cardiac action potential (Osadchii, 2017), it represents the best approximation for reliably estimating current amplitude in all our constructs (given their relatively right‐shifted *V*_50_, which resulted in small currents at lower voltages). Hence, this was the most negative voltage for which we could reliably determine current amplitude for all WT K_V_7.1/KCNE1 channels. As previously shown (Erlandsdotter et al., 2023), the concentration‐dependent reduction in *I*_40mV_ largely followed that of *G*_max_ for the human K_V_7.1/KCNE1 channel (compare Figure 1d with Figure 5a). This is expected, as 17β‐E2 only reduced the overall conductance and current for the human K_V_7.1/KCNE1 channel, without affecting *V*_50_. The same pattern was seen for the guinea pig K_V_7.1/KCNE1 channel, which was anticipated given the similar 17β‐E2 response compared to the human channel. In contrast, the zebrafish K_V_7.1/KCNE1 channel showed a more notable decrease in *I*_40mV_ (Figure 5a) than in *G*_max_. This is due to the additional shift in *V*_50_ seen for the zebrafish channel, which contributes to the current reduction at the sub‐saturating voltage of +40 mV. Expectedly, the rabbit K_V_7.1/KCNE1 channel showed a more prominent reduction of *I*_40mV_ compared to the human and guinea pig channel (Figure 5a), which is in line with an enhanced 17β‐E2 response at lower concentrations. The species variability in responding to 17β‐E2 had the largest impact at low and intermediate 17β‐E2 concentrations. At 1 µM, 17β‐E2 reduced the *I*_40mV_ of guinea pig K_V_7.1/KCNE1 by −15.4 ± 18.3% (*P* = 0.1325), zebrafish K_V_7.1/KCNE1 by −41.8 ± 10.4% (*P* = 0.0009) and rabbit K_V_7.1/KCNE1 by −73.7 ± 3.4% (*P* < 0.0001) (Figure 5b). At 3 µM, 17β‐E2 led to a reduction of the *I*_40mV_ of guinea pig K_V_7.1/KCNE1 of −28.7 ± 21.4% (*P* = 0.0068), zebrafish K_V_7.1/KCNE1 of −59.7 ± 14.9% (*P* < 0.0001) and rabbit K_V_7.1/KCNE1 of −85.4 ± 5.5% (*P* < 0.0001) (Figure 5b). Moreover, 17β‐E2 concentrations lower than 1 µM induced clear current induction for the rabbit and zebrafish channels (Figure 5a). Altogether, these data show that the different concentration dependence and magnitude of 17β‐E2 responses on K_V_7.1/KCNE1 channels from different species lead to a range of responsiveness to 17β‐E2 at a physiologically relevant voltage.

## 4 DISCUSSION

In this study, we show that 17β‐E2 inhibits the K_V_7.1/KCNE1 channel from all tested animal species (guinea pig, zebrafish and rabbit) when expressed in *Xenopus* oocytes, which is similar to what has been previously found for the human K_V_7.1/KCNE1 channel. However, the concentration dependence and mechanism of inhibition vary between species, with the rabbit channel responding to lower 17β‐E2 concentrations than the other species, and the zebrafish channel responding with an additional inhibiting *V*_50_ shift.

Variability in response to pharmacological agents by ion channels from different species has been reported previously for other ion channels and/or agents. For instance, TRPV2 and TRPA1 channels from different species respond differently to channel modulators like 2‐aminoethoxydiphenyl borate and specific benzamides (Juvin et al., 2007; Tanner & Beeton, 2018). For the K_V_7.1/KCNE1 channel, species variability has been reported in response to protein kinase C (PKC)‐mediated inhibition and linoleoyl glycine (Lin‐Gly)‐mediated activation, where the guinea pig channel shows impaired response. For PKC, this has in part been attributed to an amino acid substitution at residue S102 in KCNE1 (Abbott, 2014; Kanda et al., 2011; Varnum et al., 1993). For Lin‐Gly, this has in part been attributed to three amino acid substitutions at residues 36–38 in KCNE1 (Skarsfeldt et al., 2020). Moreover, the zebrafish K_V_7.1/KCNE1 channel has been shown to respond less to specific channel inhibitors (JNJ303) and activators docosahexaenoyl glycine (DHA‐Gly) than the human K_V_7.1/KCNE1 channel (De la Cruz et al., 2023), and to show atypical responses to channel activators like R‐L3 and mefenamic acid (Haverinen et al., 2022). Although the molecular mechanisms underlying the deviating responses to such compounds remain to be determined, the poor conservation of the human and zebrafish KCNE1 subunit and possibly altered KCNE1 association with zebrafish K_V_7.1 have been speculated to contribute (De la Cruz et al., 2023; Haverinen et al., 2022). For the species variability of K_V_7.1/KCNE1 channels to 17β‐E2, we were not able to find a specific sequence alteration that underlay the effect. Moreover, we found that the species variability in the 17β‐E2 effect was not simply determined by either the K_V_7.1 subunit or the KCNE1 subunit, but rather is determined by the K_V_7.1/KCNE1 complex of each species. Speculatively, this could hint to a 17β‐E2 mechanism of action that involves the interface between K_V_7.1 and KCNE1 subunits, and that variability in this interface (or how 17β‐E2 induces current reduction) underlies the altered concentration–response relationship of the rabbit channel, and the additional *V*_50_ response of the zebrafish channel. We concluded in our previous study (Erlandsdotter et al., 2023) that classical steroid receptor pathways through oestrogen receptor‐ and GPR30‐dependent signalling are unlikely to mediate the 17β‐E2 effects on K_V_7.1/KCNE1, as inhibiting these pathways did not impair the effect of 17β‐E2. However, a possible contribution from other signalling pathways cannot be excluded, which should be taken into consideration in future studies elucidating the mechanism of action of 17β‐E2 inhibition of this channel.

A limitation of this study is the use of ethanol as solvent for 17β‐E2, as our previous study showed that higher concentrations of 17β‐E2 are needed to induce inhibition of the human K_V_7.1/KCNE1 channel, compared to using dimethyl sulfoxide as solvent (Erlandsdotter et al., 2023). However, as ethanol as 17β‐E2 solvent has been used in several of the previous studies in cardiomyocytes and in our assessment of channel mutants (Cheng et al., 2012; Erlandsdotter et al., 2023; Kurokawa et al., 2008; Tanabe et al., 1999), we preferred this solvent in the present study. Of note, the nature of the 17β‐E2 effect is similar independent of solvent; it is just the concentration dependence of effects that vary (Erlandsdotter et al., 2023). Moreover, the 17β‐E2 concentrations used in this study and many other studies in experimental models are higher than reported plasma concentrations, which range from subnanomolar to 0.1 µM (reference ranges from the American College of Physicians and the Mayo Clinic). Another limitation is the need for caution in translating the importance of our zebrafish K_V_7.1/KCNE1 channel findings to an in vivo setting, given that the native zebrafish *I*_Ks_ likely is generated by K_V_7.1 channels with a sub‐saturating number of KCNE1 subunits co‐assembled (Abramochkin et al., 2018). Hence, reducing the number of KCNE1 subunits is expected to impair the zebrafish channel response to 17β‐E2, as has been shown for the human K_V_7.1/KCNE1 channel (Erlandsdotter et al., 2023), ultimately rendering the channel insensitive to 17β‐E2 when no KCNE1 subunits are associated, as shown in this study for zebrafish K_V_7.1 alone.

Altogether, this study provides a quantitative comparison of the effect of 17β‐E2 on the K_V_7.1/KCNE1 channel from species commonly used in cardiac electrophysiological research and relates the effects to those previously reported for the human K_V_7.1/KCNE1 channel studied in the same experimental system and under similar conditions. This work highlights variability in the inhibiting effect of 17β‐E2 on the K_V_7.1/KCNE1 channel from different species, which may be of use in interpreting translational effects of 17β‐E2 in cardiac contexts. When just considering the K_V_7.1/KCNE1 response per se, our data suggests that the guinea pig channel has a response most comparable to the human channel, whereas both the rabbit and zebrafish channels show enhanced responses. Hence, in addition to species variability in the relative contribution of the native *I*_Ks_ to cardiac repolarization (Faber & Rudy, 2000; Lu et al., 2001; O'Hara & Rudy, 2012), which will impact how prominently K_V_7.1/KCNE1 channel modulation in each species affects cardiomyocyte electrophysiology, this study suggests that also variability in the pharmacological profile of K_V_7.1/KCNE1 may be a contributing factor.

## AUTHOR CONTRIBUTIONS

Veronika A. Linhart: conception and design of the work; acquisition, analysis and interpretation of data for the work; and drafting the work and revising the work critically for important intellectual content. Lucas Dauga: design of the work; acquisition, analysis and interpretation of data for the work; and revising the work critically for important intellectual content. Sara I. Liin: conception and design of the work; interpretation of data for the work; and drafting the work and revising the work critically for important intellectual content. All authors approved the final version of the manuscript; agree to be accountable for all aspects of the work in ensuring that questions related to the accuracy or integrity of any part of the work are appropriately investigated and resolved; and all persons designated as authors qualify for authorship, and all those who qualify for authorship are listed.

## CONFLICT OF INTEREST

None declared.

## Data Availability

All data supporting the results are provided in the figures and tables.
